# A Delphi Study on Identifying Competencies in Virtual Healthcare for Healthcare Professionals

**DOI:** 10.3390/healthcare12070739

**Published:** 2024-03-29

**Authors:** Ibrahim Mubarak Al Baalharith, Ahmad Eissa Aboshaiqah

**Affiliations:** 1 College of Nursing, King Saud University, Riyadh 11421, Saudi Arabia; aaboshaiqah@ksu.edu.sa; 2 Governance and Excellence, SEHA Virtual Hospital, Riyadh 11421, Saudi Arabia

**Keywords:** virtual care, competence, competencies, education, framework, Delphi, healthcare professionals, Saudi Arabia

## Abstract

Background: Virtual care adoption accelerated during the COVID-19 pandemic, highlighting the need for healthcare professionals to develop relevant competencies. However, limited evidence exists on the core competencies required for quality virtual care delivery. Objective: This study aimed to identify the critical competencies physicians, nurses, and other health professionals need for adequate virtual care provision in Saudi Arabia using a Delphi method. Methods: A 3-round Delphi technique was applied with a panel of 42 experts, including policymakers, healthcare professionals, academicians, and telehealth specialists. In Round 1, an open-ended questionnaire elicited competencies needed for virtual care. The competencies were distilled and rated for importance in Rounds 2 and 3 until consensus was achieved. Results: Consensus emerged on 151 competencies across 33 domains. The most prominent domains were communication (15 competencies), professionalism (13), leadership (12), health informatics (5), digital literacy (5), and clinical expertise (11).

## 1. Introduction

### 1.1. Background

Virtual health has become one of the most essential approaches in the healthcare sector to assist in effective and timely healthcare service delivery. The COVID-19 pandemic presented an unprecedented challenge to healthcare professionals and patients globally [1]. They faced safety issues since the disease could be passed from one person to another through contact [2]. Consequently, the adoption of virtual care was accelerated. Virtual care refers to the remote delivery of healthcare services like telemedicine, mHealth, remote patient monitoring, and others using information and communication technologies [3]. While virtual care improves access and reduces costs, delivering quality healthcare virtually requires healthcare professionals to have specific competencies beyond traditional in-person care [4]. Leveraging virtual healthcare is especially relevant in Saudi Arabia, which faces a shortage of healthcare professionals and increasing rates of chronic conditions [5].

According to Alahmari, virtual care is a relatively new concept in many countries, including Saudi Arabia [6]. In the country, virtual healthcare is offered in virtual clinics, which have recently been introduced. Their quality differs based on different settings. Nevertheless, many people view them as the future of healthcare. In their study, Alahmari found that 87% of Saudi patients who received virtual healthcare in Saudi virtual clinics agreed to some extent that virtual clinics could replace conventional clinics [6]. While most patients involved in the study (86%) were satisfied with the services they received through the virtual clinics, concerns persist over the quality of virtual healthcare [6]. Most of these concerns are associated with healthcare professionals’ ability to transition from conventional face-to-face healthcare to virtual healthcare, often without sufficient training, as was experienced during the COVID-19 pandemic [2].

The literature has continuously proven the increasing number of facilities incorporating virtual health into their system. This aligns with the implications that virtual health will emanate when health practitioners are well skilled and equipped with competency-based practices [7]. Health practitioners need these skills and experiences to provide high-quality treatment, effectively use communication equipment to communicate and analyze patients’ data, and understand the requirements of virtual healthcare delivery. In addition, “Competencies extend to the administrative aspects of virtual care delivery and the cultivation of positive relationships with patients and their families” [8].

Moreover, there is a dearth of research on the core competencies healthcare professionals need for effective virtual care delivery. Identifying these competencies can inform the development of training programs and guidelines to equip healthcare professionals with the skills required for high-quality virtual healthcare delivery [9]. It can also help Saudi Arabia and other governments worldwide when integrating virtual healthcare as part of their strategies to expand healthcare to their citizens.

Saudi Arabia is one of the countries that have embraced digital health. The Saudi Arabian government currently supports the mainstreaming of digital health. The government of Saudi Arabia has been using technology extensively to promote healthcare service delivery. For instance, after the outbreak of COVID-19, the government of Saudi Arabia started supporting public health precautions to control its spread [10]. Some of the digital health systems that the government has used to track COVID-19 patients are Tabaud, Seha, and Tetamman. Jonasdottir et al. state, “This network was formed to use virtual healthcare and telehealth solutions healthcare facilities to connect with primary healthcare centres and hospitals operating in remote locations” [11]. These applications have helped in linking healthcare practitioners with patients and, in addition, reduce the need for patients to visit healthcare facilities. According to MOH records, Saudi Arabia had more than two million virtual healthcare service users as of 2022 [12].

Saudi Arabia has launched the world’s largest virtual hospital of its kind. SEHA Virtual Hospital (SVH) is the first virtual hospital in the Middle East and North Africa and the first hospital worldwide to obtain Canadian accreditation. It offers more than 29 specialties and 73 subspecialties [13]. It is connected to a network of over 170 hospitals across all regions in Saudi Arabia. In these hospitals, patients can attend real-time video sessions with the country’s top specialists, who provide their required care. They share their vital signs, while tests and x-rays are shared with a network of specialists who offer their assessments and healthcare recommendations. The hospital’s specialists provide multiple healthcare services, including emergence and critical advice (such as virtual strokes and electroencephalography), specialized clinics (such as blood diseases, psychiatry, and heart diseases), home care services, and medical support services (including virtual pharmacy services and virtual pathology) [13].

In addition, to enhance remote patient contact, the Saudi Commission for Health Specialties (SCFHS) introduced the telemedicine training program: “The government has also supported digitizing Healthcare, especially promoting virtual healthcare adoption and continued use” [11]. According to Fronczek and Rouhana [14], the Ministry of Health in Saudi Arabia developed a strategy to improve the quality and efficiency of healthcare services [15].

#### Aim of the Study

This study’s objective is to close the research gap on healthcare professionals’ competencies in virtual health. It seeks to answer the following research question:

RQ: What competencies (knowledge, skills, and attitudes) are required for healthcare professionals to provide quality virtual healthcare to patients?

Its specific objectives are as follows:

1. To identify the core competencies required by physicians, nurses, and allied healthcare professionals in Saudi Arabia to deliver quality virtual care effectively.
2. To understand Saudi healthcare professionals’ challenges in adopting virtual care and applying related competencies.
3. To recommend how training programs and licensing requirements could be enhanced to develop virtual care competencies amongst Saudi healthcare professionals.
4. To suggest organizational and technological supports to enable Saudi healthcare professionals to integrate virtual care competencies into routine practice.

## 2. Materials and Methods

### 2.1. Research Design

The Delphi method was used for this study, which combined both quantitative and qualitative elements, which helped obtain a consensus from experts that was reliable enough to create a comprehensive framework. This method is structured iteratively to gather expert opinions and reach a consensus on a complex problem or issue. It involves a series of rounds of data collection and analysis.

#### 2.1.1. Study Setting and Participant Selection

The study was conducted at SEHA Virtual Hospital in Saudi Arabia. A purposeful sampling technique was used to choose experts based on their quality, knowledge, experience regarding virtual health, and clinical and academic credentials. The study involved 42 participants. These participants were all staff members of the SEHA hospital. The inclusion criterion was having some experience providing virtual healthcare services. Selected participants were requested to complete each round of the Delphi survey within 2–3 weeks of receiving the email for each round. A reminder email was sent to non-responders three days before the deadline to prompt their completion of the survey. Communication between the researchers and the panelists was via email only so that no expert could exert an undue influence over the opinions of others. Participants were known to the researchers but were not known to the other participants to maintain anonymity among the participants.

#### 2.1.2. Data Collection and Materials

The participants’ responses were collected from online questionnaires. They responded to the questionnaire online. The questionnaires prompted the participants to provide qualitative and quantitative data.

#### 2.1.3. Validity and Reliability of the Questionnaire

Nevertheless, there is a lack of proof regarding the dependability of the Delphi method, as indicated by Watson et al. in 2008 [16]. 

The validity of the questionnaire was determined by putting it through a thorough validation process in order to prove its content validity. The items on the questionnaire were subjected to thorough reviews and evaluations by expert panels, which comprised subject-matter specialists, to determine whether or not they were relevant, clear, and appropriate. In order to guarantee that the questionnaire effectively evaluated the targeted components connected to virtual healthcare abilities, their comments were painstakingly included in the final version of the questionnaire. Regarding the reliability of the questionnaire, we carried out stringent tests to evaluate the consistency and stability of the measurements over time. This was performed in order to determine the reliability of the questionnaire. When determining the reliability of the questionnaire items, internal consistency metrics, such as Cronbach’s alpha, were utilized as part of the evaluation process.

#### 2.1.4. Procedure

The Delphi method was used to collect participants’ responses. It involved three rounds. The first questionnaire prompted participants to provide qualitative data. Participants were asked open-ended questions that required them to showcase their knowledge, skills, and attitudes regarding virtual health. They were asked to identify up to ten competencies that they believed healthcare professionals need. Participants were also required to provide their demographic data.

In the first round, a questionnaire was anonymously sent to a panel of experts, seeking their views on the issue. Then, the researchers summarized the responses and returned them to the experts for a second round. The scholars could make changes in every round of the experiment [17], rating and ranking the items using the changes made in each round. This procedure of iterations persisted until a pact was reached. Different justifications prompted choosing this methodology for the research. For instance, it simplified the collection of expert opinions from a very diverse group of respondents located in other geographic areas with the aid of a questionnaire and thereby reduced the need for face-to-face gatherings.

Moreover, the respondents remained anonymous during the entire study and therefore freely spoke about what they believed were key matters for issues of the study. Thus, the Delphi technique allowed for several rounds of fine-tuning and responding to the group results; experts appeared ready and then reacted in a conducive fashion, resulting in consensus. This method was also focused on anonymity and allowed experts to share their true viewpoints without being subjected the influence of any external factors or corresponding pressures from co-panelists [18]. Boulkedid, found that it also included structured feedback conjointly with multiple rounds for experts to refine their opinions based on social results, thus developing an agreement [19].

Therefore, the study procedure produced reliable and realizable findings. Last, given the promulgated method, which followed a structured path, it gave an audit trail indicating how conclusions were attained. Consequently, the study could be audited to ensure that its findings are credible and applicable.

## 3. Results

This study was conducted to determine the competency requirements for healthcare professionals who work in virtual healthcare settings. Experts were part of a panel, going through three rounds of discussion to arrive at an agreement on key items.

### 3.1. Sociodemographic and Professional Characteristics of the Expert Panel

Forty-two panelists participated in all three rounds. The panelists included nurses, physicians, pharmacists, radiologists, and social workers. Most panelists (42.5%) had a master’s degree, while 21.4% had a bachelor’s degree. Most panelists (66.7%) had 10–19 years of clinical experience. However, only 4.8% had over ten years of experience in virtual care, with most of them (64.3%) having 1–4 years of experience. Table 1 provides an overview of the sociodemographic and professional characteristics of the Delphi expert panel.

### 3.2. Delphi Round 1

The first-round Delphi technique questionnaire was qualitative in nature and consisted of open-ended questions requesting panel members to identify knowledge, skills, and attitudes for virtual health. This round was essential for the research to help initiate an initial understanding of the three aspects of competence needed by every health professional in the healthcare setting. Participants were asked to identify up to 10 competencies that healthcare professionals must have to work in virtual health. Responding to the open-ended prompt questions in Round 1, participants identified 227 types of knowledge and 121 skills that healthcare professionals should have. They also showed 72 different types of attitudes regarding virtual healthcare. After qualitative content analysis, 33 domains and 157 items were generated from the panelists after reviews from the research supervisor and an international expert in the field.

The response statements generated in Round 1 have been excluded from this manuscript in order to focus on the competency findings.

### 3.3. Delphi Round 2

The domains and items derived from the initial questionnaire were utilized in formulating the second-round Delphi questionnaire. Panel members were required to express their level of agreement or disagreement using a five-point Likert scale for each item. Moreover, experts were asked to add items that they believed to be important for healthcare professionals to be competent in virtual health but had yet to be included in the list. The experts demonstrated strong agreement with all 33 domains items. 

They agreed on 157 items, with a mean ≥ 4.40 and S.D. ≤ 1.02. Moreover, 14 new, non-duplicated items emerging from the open-ended data were identified, including C8, C11, C14, C53, C66, C67, C68, C69, C83, C90, C128, C129, C131, and C164 (Table S1 (Supplementary Materials)). Therefore, a list of 165 items in 33 domains was sent to 42 experts for the third survey in Round 3. The researchers computed the Cronbach’s alpha coefficient for measuring reliability. The Cronbach’s alpha based on the standardized items was 0.942 in this round. Cronbach’s alpha coefficients range from 0 to 1, and values greater than 0.7 reflect acceptable reliability [20].

### 3.4. Delphi Round 3

In Round 3, all 165 competency items reached consensus, which was defined as ≥95% agreement on importance. The agreement increased from Rounds 2 to 3 on almost all items, indicating that the Delphi process helped build consensus. For example, item C2 went from a mean of 4.29 (SD: 1.02) in Round 2 to 4.93 (SD: 0.26) in Round 3, with agreement increasing from 83% to 99%. Certain domains, like technology proficiency, professionalism, clinical expertise, communication, and teamwork, had high levels of consensus (95–100% agreement) on most competencies. Other domains, like health equity, public health, and billing had more variation, with some items not reaching 95% agreement. The Cronbach’s alpha based on the standardized items was 0.930 in this round.

A final list of 165 competencies in 33 domains was finalized, as shown in Table S2 (Supplementary Materials).

### 3.5. External Validation

Four external reviewers were invited to validate the importance, relevance, and comprehensiveness of the study’s results. The reviewers included international experts in virtual care and did not participate in this study and were recommended by experts based on their reputations in the field of virtual health. The reviewers agreed that all items were adequately covered, and the results were applicable in different virtual care settings.

## 4. Discussion

In this study, we established a set of competencies for health professionals in Saudi Arabia through a three-round Delphi survey, which identified a series of knowledge, skills, and other abilities essential to achieving virtual health. This consensus-based set of competencies provides the first comprehensive description of the competency frameworks in Saudi health systems for healthcare professionals to work effectively in virtual health settings. In our study, one hundred and sixty-five competencies were identified as highly important for health professionals who work on virtual health (Supplementary Materials). The competencies were in agreement, ranging from 94.29% to 100%. This agreement was brought about by the mean and SD of Round 3, where the mean went from 4 to 5 while the SD, on the other hand, ranged from 0.00. to 0.64. In all the domains, as showcased in Table S2 (Supplementary Materials), the competencies required the health professionals to understand and know the specific domain. In addition, they had to have the ability to navigate through the virtual healthcare system, including the software involved. Equally important, organizational culture needs to embrace virtual care as a legitimate model of quality care, at par with traditional in-person delivery [21].

This study provides a framework for various stakeholders, from healthcare administrators to educators, to understand the competencies needed in an increasingly digital healthcare world. This ensures a more integrated and effective approach to virtual healthcare services, benefiting patients through improved quality and safety. While the Saudi Commission provides regulation and guidance for health specialists and the Ministry of Health (MOH), the results from this study can be used to develop comprehensive rules and guidelines for virtual health practice in Saudi Arabia. A clear framework will ensure that healthcare professionals have the competencies to deliver high-quality virtual healthcare [22]. Moreover, one of the main goals of healthcare is to offer safe and high-quality service. Identifying the right competencies ensures that healthcare providers are well equipped to provide this level of care, even in a virtual setting [23]. Moreover, universities and educational institutions can use experiential learning through simulations and telehealth encounters to build healthcare professionals’ competency [24].

Virtual health development has brought rise to the need for health professionals who are skilled with the needed competencies to surpass the barriers associated with virtual healthcare and provide high-quality patient care. Digital technology proficiency in virtual healthcare (C1–C5) and artificial intelligence ensures high-quality healthcare delivery and positive patient outcomes [25]. Without digital competence, there will be barriers to navigating telehealth systems, leading to disparities in healthcare access and outcomes [26]. Competencies related to professionalism (C6–C14) and ethical considerations (C35–C40) also play an important role.

When health practitioners are both professional and have ethical considerations, health information management skills (C97–C102), and data security and privacy (C103–C107), then healthcare is “guaranteed to have quality of care, sustainable costs, professional liability and respect of patient privacy, and data protection and confidentiality” [27]. Clinical expertise and decision-making (C15–C21) allow “healthcare providers to have the knowledge and ability to determine when virtual care is appropriate” [28]. According to HRRS, health equity in virtual care (C22–C25) and virtual health leadership and management (C26–C34) competencies will help health practitioners understand the role of virtual healthcare and aid in meeting the needs of marginalized communities [29]. Professionals must also have a sense of collaboration and teamwork (C41–C46) for improved performance [12].

Similarly, care coordination and integration of virtual healthcare (C47–C53) are the common competencies needed for effective workflow and also to ensure that timely and coordinated care is received by patients [30]. Innovature BPO, states that it is easier to make effective decisions when one has competence in data utilization (C58–C62) [31]. In contrast, cultural competency (C54–C57) allows practitioners to care for a diverse population [9]. A proper recordkeeping and documentation routine in virtual healthcare (C70–C74) is essential for ensuring safe continuity of care [32]. Recognition of diseases and their specific management (C63–C69) and patient safety competencies (C121–C123) guarantee that the patient’s care is safe [10].

Effective communication (C75–C80) and emergency response and crisis management (C81–C87) competencies in virtual healthcare are important aspects of ensuring collaborative teamwork and fostering safe patient care [33]. Similarly, practitioners with competencies in research development and evidence-based practice guarantee high-quality healthcare outcomes [13]. In addition, “Digital marketing will play a role in promoting telehealth services and educating patients and society about its benefits” [8]. Therefore, health practitioners need to be competent in marketing and outreach. When patients adapt to innovative technologies (C147–C149) and engage in continuous learning and development (C108–C114), their skills are expounded and advanced, and advancement in their careers is witnessed [34].

Practitioners with patient care assessment and diagnosis (C121–C123) and patient-centered care (C124–C128) competencies tend to make decisions based on patient preference, as they are only subjected to the patient’s needs [35]. Project management is important because it ensures compliance with ethical and legal policies [36]. Remote patient monitoring (C134–C138) and remote medication management competency (C129–C133) will reduce acute hospital events [37]. Similarly, health practitioners who can perform correct billing and coding impact virtual health performance since “Incorrect billing and coding can result in denied claims or delayed and decreased payments” [38]. According to Javaid, virtual health must employ workers with cybersecurity competence for better protection of the information of the patients [39]. All the competencies of C165 are essential in ensuring that the health outcomes for patients are improved.

### 4.1. Recommendations and Conclusions

This study’s findings are crucial for enhancing healthcare delivery in an increasingly digitalizing world. They lay out an extensive list containing 165 competencies across 33 domains. These competencies provide a strong foundation for training, regulation, and virtual care practice in Saudi Arabia and other countries. Several key recommendations emerge from the results, as discussed below.

Virtual care competencies should be integrated into both undergraduate and postgraduate medical curricula in Saudi Arabia. Universities and educational institutions must equip future healthcare professionals with knowledge and skills for virtual practice. They can achieve this objective by incorporating relevant competencies into telehealth, informatics, communication, ethics, and clinical care courses. For current healthcare practitioners, the Saudi Commission for Health Specialties, which oversees licensing, can mandate certified virtual care training programs that teach the identified competencies. Healthcare professional associations can also design continuing medical education activities to develop virtual care competencies among their members.

The study provides a starting point for governments to develop a standardized national virtual care competency framework to guide the training and certification of healthcare professionals. Multiple stakeholders in the healthcare sector should adapt and validate the study’s findings to create nation-specific frameworks similar to those established in the United States and Canada. The frameworks can define competency domains, specific skills, and performance metrics. Certifications can then be offered by providers to validate competency attainment.

Virtual care technologies should be designed to enable and enhance competent practice. Platforms should embed features like intuitive interfaces, clinical prompts, smart templates, and analytics dashboards. This allows providers to seamlessly apply competencies as they interact with the system. Extensive training is key so that professionals will maximize these functionalities for quality care delivery.

Healthcare organizations also play a critical role in competency integration. Supportive policies, incentives, and culture must be cultivated to drive the adoption of virtual modalities into routine practice. Policies can promote the use of telehealth for communication, consultations, monitoring, and team collaboration. Incentives can encourage training, certification, and ongoing development.

### 4.2. Limitations

Despite the fact that the current study observes and describes progress in regard to recognizing and describing healthcare competencies which are relevant for virtual healthcare, it is nevertheless imperative to engage with some limitations affecting the applicability of the study findings. Data obtainment was restricted by the diversity limitations of the participant group, who were all healthcare professionals from SEHA’s Virtual Hospital, which is proclaimed to be the largest hospital in the world and stands as the exclusive virtual hospital in Saudi Arabia. This study encounters an additional limitation due to the small panel size, even though it is within the recommended range for Delphi studies.

## Figures and Tables

**Table 1:** Sociodemographic and professional characteristics of the expert panel.

Characteristic	Round 1 N (%)	Round 2 N (%)	Round 3 N (%)
Gender			
Male	21 (50%)	21 (50%)	21 (50%)
Female	21 (50%)	21 (50%)	21 (50%)
Profession			
Nurse	19 (45.2%)	19 (45.2%)	19 (45.2%)
Physician	14 (33.3%)	14 (33.3%)	14 (33.3%)
Pharmacist	3 (7.1%)	3 (7.1%)	3 (7.1%)
Radiology	3 (7.1%)	3 (7.1%)	3 (7.1%)
Social Worker	3 (7.1%)	3 (7.1%)	3 (7.1%)
Nationality			
Saudi	40 (95.2%)	40 (95.2%)	40 (95.2%)
Non-Saudi	2 (4.8%)	2 (4.8%)	2 (4.8%)
Current Position			
Manager/Supervisor	24 (57.1%)	24 (57.1%)	24 (57.1%)
Educator	9 (21.4%)	9 (21.4%)	9 (21.4%)
Researcher	3 (7.1%)	3 (7.1%)	3 (7.1%)
Staff	6 (14.3%)	6 (14.3%)	6 (14.3%)
Qualification			
Bachelor’s Degree	9 (21.4%)	9 (21.4%)	9 (21.4%)
Master’s Degree	19 (45.2%)	19 (45.2%)	19 (45.2%)
Doctoral Degree	7 (16.7%)	7 (16.7%)	7 (16.7%)
Fellowship	5 (11.9%)	5 (11.9%)	5 (11.9%)
Board Certificate	2 (4.8%)	2 (4.8%)	2 (4.8%)
Clinical Experience			
10–14 Years	15 (35.7%)	15 (35.7%)	15 (35.7%)
15–19 Years	13 (31%)	13 (31%)	13 (31%)
20–24 Years	12 (28.6%)	12 (28.6%)	12 (28.6%)
5–9 Years	2 (4.8%)	2 (4.8%)	2 (4.8%)
Virtual Health Experience			
1–4 Years	27 (64.3%)	27 (64.3%)	27 (64.3%)
5–9 Years	13 (31%)	13 (31%)	13 (31%)
10–14 Years	2 (4.8%)	2 (4.8%)	2 (4.8%)

## Data Availability

All data generated or analyzed during this study are included in this published article. Additional information is available from the corresponding author on reasonable request.

## Supplementary Materials

The following supporting information can be downloaded at https://www.mdpi.com/article/10.3390/healthcare12070739/s1, Table S1: Round 2 and 3 results; Table S2: Final list of competencies from Delphi method.
