# Supplementary material for: A Delphi Study on Identifying Competencies in Virtual Healthcare for Healthcare Professionals
Source: Healthcare (Basel). 2024 Mar 29;12(7):739. doi: 10.3390/healthcare12070739 (PMC11011667; doi:10.3390/healthcare12070739)
Supplement: Supplementary file 1 [file healthcare-12-00739-s001.zip › healthcare-2896766-supplementary.pdf]

Supplementary Materials

Table S1. Round 2 and 3 results

Domain	Items	Round 2		Round 3		
		Mean	SD	Mean	SD	Agreement (%)
**Domain1.** Digital technology Proficiency	C1	4.48	0.59	4.95	0.22	99.05
	C2	4.29	1.02	4.93	0.26	98.57
	C3	4.88	0.45	4.93	0.26	98.57
	C4	4.81	0.40	4.86	0.35	97.14
	C5	4.79	0.42	4.93	0.26	98.57
**Domain2.** Professionalism in Provision of Virtual Care	C6	5.00	0.00	4.98	0.15	99.52
	C7	4.40	0.59	4.95	0.22	99.05
	C8	-	-	4.93	0.26	98.57
	C9	5.00	0.00	4.95	0.22	99.05
	C10	4.81	0.40	4.95	0.22	99.05
	C11	-	-	4.98	0.15	99.52
	C12	4.50	0.63	4.95	0.22	99.05
	C13	4.64	0.48	4.98	0.15	99.52
	C14	-	-	4.95	0.22	99.05
**Domain3.** Clinical Expertise and decision making	C15	5.00	0.00	4.95	0.22	99.05
	C16	5.00	0.00	5.00	0.00	100.00
	C17	4.83	0.38	4.95	0.22	99.05
	C18	4.93	0.34	4.95	0.22	99.05
	C19	4.90	0.37	4.98	0.15	99.52
	C20	4.86	0.42	4.95	0.22	99.05
	C21	4.50	0.63	4.98	0.15	99.52
**Domain4.** Health Equity in Virtual Care	C22	4.48	0.59	4.71	0.64	94.29
	C23	5.00	0.00	4.71	0.60	94.29
	C24	5.00	0.00	4.83	0.44	96.67
	C25	4.50	0.63	4.81	0.55	96.19
**Domain5.** Virtual Health Leadership and Management	C26	4.88	0.45	5.00	0.00	100.00
	C27	5.00	0.00	4.90	0.30	98.10
	C28	4.36	0.66	4.98	0.15	99.52
	C29	4.79	0.42	5.00	0.00	100.00
	C30	4.79	0.42	4.95	0.22	99.05
	C31	5.00	0.00	4.93	0.26	98.57
	C32	4.38	0.66	4.98	0.15	99.52
	C33	5.00	0.00	4.98	0.15	99.52
	C34	4.52	0.71	5.00	0.00	100.00
**Domain6.** Legal and Ethical Considerations in Virtual Health	C35	4.81	0.40	4.98	0.15	99.52
	C36	4.76	0.43	4.98	0.15	99.52
	C37	5.00	0.00	4.98	0.15	99.52
	C38	4.52	0.71	4.98	0.15	99.52
	C39	4.64	0.48	4.98	0.15	99.52
	C40	4.52	0.71	4.98	0.15	99.52
**Domain7.** Teamwork and Collaboration in virtual healthcare	C41	4.90	0.37	4.98	0.15	99.52
	C42	4.93	0.34	4.98	0.15	99.52
	C43	4.86	0.35	4.98	0.15	99.52
	C44	5.00	0.00	4.95	0.22	99.05
	C45	4.98	0.15	4.98	0.15	99.52
	C46	4.50	0.71	4.98	0.15	99.52
**Domain8.**	C47	5.00	0.00	4.98	0.15	99.52

Care Coordination and Integration of virtual healthcare	C48	4.29	1.02	4.95	0.22	99.05
	C49	4.88	0.45	4.98	0.15	99.52
	C50	5.00	0.00	5.00	0.00	100.00
	C51	4.40	0.66	4.93	0.26	98.57
	C52	4.50	0.63	5.00	0.00	100.00
	C53	-	-	4.95	0.22	99.05
**Domain9.** Cultural competency in virtual healthcare	C54	5.00	0.00	4.98	0.15	99.52
	C55	4.43	0.63	4.95	0.22	99.05
	C56	4.55	0.67	4.93	0.26	98.57
	C57	4.71	0.46	4.98	0.15	99.52
**Domain10.** Data Analytics and Interpretation in virtual healthcare	C58	4.83	0.44	4.95	0.22	99.05
	C59	4.76	0.43	4.93	0.26	98.57
	C60	5.00	0.00	4.98	0.15	99.52
	C61	4.50	0.63	4.93	0.26	98.57
	C62	4.79	0.42	4.98	0.15	99.52
**Domain11.** Disease - Specific Management in virtual healthcare	C63	4.64	0.48	4.98	0.15	99.52
	C64	4.95	0.22	5.00	0.00	100.00
	C65	4.95	0.22	4.95	0.22	99.05
	C66	-	-	4.98	0.15	99.52
	C67	-	-	4.98	0.15	99.52
	C68	-	-	4.98	0.15	99.52
	C69	-	-	4.95	0.22	99.05
**Domain12.** Recordkeeping and Documentation in virtual healthcare	C70	4.88	0.33	4.98	0.15	99.52
	C71	4.98	0.15	4.95	0.22	99.05
	C72	4.74	0.45	5.00	0.00	100.00
	C73	5.00	0.00	4.98	0.15	99.52
	C74	4.48	0.59	4.95	0.22	99.05
**Domain13.** Effective Communication in virtual healthcare	C75	4.29	1.02	4.98	0.15	99.52
	C76	5.00	0.00	4.93	0.26	98.57
	C77	5.00	0.00	4.98	0.15	99.52
	C78	4.81	0.40	4.93	0.26	98.57
	C79	4.50	0.63	4.98	0.15	99.52
	C80	5.00	0.00	4.98	0.15	99.52
**Domain14.** Emergency response and crisis management in virtual healthcare	C81	4.98	0.15	4.98	0.15	99.52
	C82	4.12	0.86	4.98	0.15	99.52
	C83	-	-	4.88	0.33	97.62
	C84	4.76	0.43	4.98	0.15	99.52
	C85	4.79	0.42	4.93	0.26	98.57
	C86	4.50	0.63	4.98	0.15	99.52
	C87	4.64	0.48	4.98	0.15	99.52
**Domain15.** Research development and Evidence-Based Practice Utilization in virtual healthcare	C88	4.98	0.15	5.00	0.00	100.00
	C89	4.86	0.35	4.95	0.22	99.05
	C90	-	-	4.95	0.22	99.05
	C91	4.90	0.37	4.95	0.22	99.05
**Domain16.** Governance, risk and quality management in virtual healthcare	C92	4.76	0.43	4.98	0.15	99.52
	C93	5.00	0.00	4.98	0.15	99.52
	C94	5.00	0.00	4.95	0.22	99.05
	C95	4.50	0.63	5.00	0.00	100.00
	C96	4.79	0.52	4.95	0.22	99.05
**Domain17.** Health Information Management in virtual healthcare	C97	4.81	0.40	4.95	0.22	99.05
	C98	5.00	0.00	5.00	0.00	100.00
	C99	4.36	0.88	4.93	0.26	98.57
	C100	5.00	0.00	4.95	0.22	99.05

	C101	4.50	0.63	4.95	0.22	99.05
	C102	5.00	0.00	4.95	0.22	99.05
**Domain18.** Data security and privacy in virtual healthcare	C103	4.81	0.40	5.00	0.00	100.00
	C104	4.79	0.42	4.95	0.22	99.05
	C105	4.74	0.50	4.98	0.15	99.52
	C106	4.50	0.63	5.00	0.00	100.00
	C107	4.40	0.59	5.00	0.00	100.00
**Domain19.** Professional Development in virtual healthcare	C108	4.12	0.86	4.98	0.15	99.52
	C109	4.81	0.40	4.98	0.15	99.52
	C110	4.76	0.43	5.00	0.00	100.00
	C111	5.00	0.00	4.95	0.22	99.05
	C112	4.52	0.71	4.98	0.15	99.52
	C113	4.52	0.71	4.98	0.15	99.52
	C114	4.50	0.63	5.00	0.00	100.00
**Domain20.** Patient Assessment and Diagnosis in virtual healthcare	C115	5.00	0.00	4.98	0.15	99.52
	C116	5.00	0.00	4.95	0.22	99.05
	C117	4.50	0.63	4.95	0.22	99.05
	C118	4.86	0.35	4.98	0.15	99.52
**Domain21.** Patient education and engagement in virtual healthcare	C119	4.50	0.63	5.00	0.00	100.00
	C120	4.90	0.37	5.00	0.00	100.00
**Domain22.** Patient Safety in virtual healthcare	C121	4.76	0.43	5.00	0.00	100.00
	C122	4.50	0.63	4.95	0.22	99.05
	C123	5.00	0.00	4.98	0.15	99.52
**Domain23.** Patient Centered Care in virtual healthcare	C124	5.00	0.00	5.00	0.00	100.00
	C125	4.40	0.66	4.98	0.15	99.52
	C126	4.79	0.42	4.90	0.30	98.10
	C127	4.79	0.42	4.95	0.22	99.05
	C128	-	-	4.98	0.15	99.52
**Domain24.** Remote Medication Management	C129	-	-	5.00	0.00	100.00
	C130	4.81	0.40	5.00	0.00	100.00
	C131	-	-	5.00	0.00	100.00
	C132	4.50	0.63	4.98	0.15	99.52
	C133	5.00	0.00	5.00	0.00	100.00
**Domain25.** Remote Patient Monitoring	C134	4.79	0.42	4.98	0.15	99.52
	C135	4.50	0.63	4.98	0.15	99.52
	C136	4.67	0.48	4.93	0.26	98.57
	C137	4.50	0.63	4.95	0.22	99.05
	C138	4.86	0.35	4.98	0.15	99.52
**Domain26.** Public and community Health in virtual healthcare	C139	4.74	0.50	5.00	0.00	100.00
	C140	4.40	0.59	4.98	0.15	99.52
	C141	4.38	0.66	4.95	0.22	99.05
	C142	5.00	0.00	4.95	0.22	99.05
	C143	4.50	0.63	5.00	0.00	100.00
	C144	4.71	0.46	4.98	0.15	99.52
**Domain27.** The Use of Artificial Intelligence	C145	4.50	0.63	5.00	0.00	100.00
	C146	4.76	0.43	4.98	0.15	99.52
**Domain28.** Innovation and Creativity in virtual healthcare	C147	4.52	0.71	5.00	0.00	100.00
	C148	4.50	0.63	5.00	0.00	100.00
	C149	5.00	0.00	5.00	0.00	100.00
**Domain29.**	C150	4.50	0.63	5.00	0.00	100.00

Project Management in virtual healthcare	C151	5.00	0.00	5.00	0.00	100.00
**Domain30.** Marketing and Outreach of Virtual Health	C152	5.00	0.00	4.93	0.26	98.57
	C153	4.50	0.63	4.95	0.22	99.05
	C154	4.50	0.63	4.88	0.33	97.62
	C155	5.00	0.00	5.00	0.00	100.00
**Domain31.** Virtual Health Policy Development and Advocacy	C156	5.00	0.00	5.00	0.00	100.00
	C157	5.00	0.00	4.98	0.15	99.52
	C158	4.50	0.63	5.00	0.00	100.00
	C159	5.00	0.00	4.98	0.15	99.52
**Domain32.** Billing and coding in virtual healthcare	C160	5.00	0.00	5.00	0.00	100.00
	C161	4.50	0.63	4.93	0.26	98.57
	C162	5.00	0.00	4.98	0.15	99.52
**Domain33.** Cybersecurity in virtual healthcare	C163	5.00	0.00	4.98	0.15	99.52
	C164	-	-	4.93	0.26	98.57
	C165	4.50	0.63	4.95	0.22	99.05

Table S2. Final List of Competencies from Delphi method

Domain. 1	**Digital technology Proficiency**	
	C1.	Demonstrates knowledge of technologies, platforms, apps, and equipment used in virtual healthcare delivery, such as videoconferencing tools
	C2.	The ability to use virtual healthcare software, apps, and other digital tools to facilitate virtual consultations, share patient information securely, and troubleshoot technical issues that may arise during virtual health sessions
	C3.	The ability to continuously learn and adapt to new virtual healthcare technologies and practices
	C4.	Empathy towards patients in virtual settings, recognizing the potential of virtual health in healthcare delivery.
	C5.	Positive and proactive attitude towards technological advancements and emerging technologies, recognizing their potential to enhance healthcare delivery in virtual settings.
Domain. 2	**Professionalism in the Provision of Virtual Care**	
	C6.	It is understanding the differences between delivering in-person versus virtual health care, including the risks and benefits.
	C7.	Understanding virtual healthcare protocols, standards, guidelines, and regulations is essential for ensuring compliance and delivering safe and effective virtual healthcare services.
	C8.	Understanding of virtual health care scope of practice and expertise
	C9.	They are utilizing the virtual care platform as the appropriate method to facilitate patient-centered care that appreciates the patient's and their family's goals, concerns, beliefs, and cultural diversity.
	C10.	The ability to demonstrate professionalism and respect for the individual and ensure the virtual environment is professional and free from distractions. This includes having a clean and tidy background, dressing appropriately, and using appropriate language.
	C11.	Assume responsibility and accept accountability for professional decisions in virtual healthcare.
	C12.	Showing high levels of professionalism, including maintaining a professional environment and the use of personal and clinical practice skills
	C13.	Expressing desire and capability in usability of virtual health design to educate, mentor, and professionally support their colleagues
	C14.	Demonstrate a commitment to high-quality virtual care of their patients.
Domain. 3	**Clinical expertise and decision-making**	
	C15.	Demonstrate extensive knowledge of clinical guidelines, best practices, and comprehensive background in medical conditions, including symptoms, diagnosis, and treatment options.
	C16.	Analyze symptoms, interpret data, and diagnose accurately in a virtual setting.
	C17.	Extensive clinical knowledge and experience in their specialty to provide accurate and effective support
	C18.	The ability to make informed clinical decisions based on virtual assessments of patient data and think critically to analyze complex health issues
	C19.	The ability to apply the knowledge of primary clinical care processes, workflow, and technologies for analysis, design, Development, and implementation of health applications and information systems.
	C20.	The ability to perform clinical assessments, diagnose conditions, and provide treatment plans through virtual platforms
	C21.	Express higher levels of professionalism with comprehensive knowledge of the medical field.
Domain. 4	**Health Equity in Virtual Care**	
	C22.	Understanding the social determinants of health and non-medical factors that influence health outcomes, such as education, income, housing, and access to transportation

	C23. The ability to tailor virtual health interventions to address specific needs and reduce disparities among diverse populations in virtual settings
	C24. We emphasize the importance of equitable access to virtual health services and the active pursuit of health equity goals.
	C25. An advocacy-oriented attitude, recognizing the importance of actively working towards health equity and reducing disparities in virtual health practices.
Domain. 5	**Virtual Health Leadership and Management**
	C26. I understand leadership principles and strategies applicable to virtual health settings.
	C27. Knowledge of the structure and functioning of healthcare systems, including relevant policies and regulations, will help you navigate the virtual health landscape effectively.
	C28. Knowledge of effective time management ensures that team members can prioritize tasks, set goals, and meet deadlines.
	C29. Understanding change management principles and strategies involves managing resistance, creating buy-in, and fostering a culture of innovation and continuous improvement.
	C30. The ability to guide teams, foster innovation, and lead change in virtual health environments.
	C31. Serve as thought leaders and expert consultants within their own and other professions, contributing to advancing therapeutic interventions, practice development, and service delivery models.
	C32. Understand the team members' roles and responsibilities in virtual care.
	C33. Ability to acknowledge the need for transformative leadership to shape the future of virtual health and, in addition, enhance healthcare delivery.
	C34. We recognize the need for flexible leadership to address barriers and focus on leading the teams in virtual health settings.
Domain. 6	**Legal and Ethical Considerations in Virtual Health**
	C35. Understanding that virtual health delivery needs to follow the legal, professional, and ethical standards that guide them
	C36. Ability to acknowledge the principles of informed consent, assessing consent from patients before their engagement in virtual health services, and ensuring they know the risks, benefits, and limitations involved
	C37. Ability to adhere to ethical principles when delivering virtual health services
	C38. The ability to recognize the importance of ethical and professional standards when working with patients and colleagues in a virtual environment
	C39. Recognize the need to use Ethical decision-making skills during dilemma situations in virtual health contexts.
	C40. Ability to promote trust and integrity in virtual healthcare practices.
Domain. 7	**Teamwork and Collaboration in Virtual Healthcare**
	C41. The ability to use tools such as project management software, online platforms for communication, and document-sharing systems assists in enhancing collaboration among team members.
	C42. Recognizing the need to convey ideas, active listening skills, and analyzing feedback helps to provide an efficient work environment for team members.
	C43. Understanding of the need for emotional intelligence and how it helps to build strong relationships and understanding among team members' emotions
	C44. Recognizing the need to work effectively as part of a healthcare team and respecting the expertise and contributions of others
	C45. Recognizing the need for Listening attentively to others and also seeking clarification when necessary to enhance collaboration among the team members

	C46. Respect for interdisciplinary perspectives, fostering a positive attitude towards collaborative patient care in virtual healthcare.
Domain. 8	**Care Coordination and Integration of Virtual Healthcare**
	C47. Knowledge of care coordination and transition workflows within healthcare organizations and across different care settings
	C48. Understanding the roles and responsibilities of various healthcare professionals involved and how they collaborate to ensure smooth transitions and continuity of care in virtual healthcare.
	C49. Understanding the process of transitioning patients between different levels and settings of care and knowledge of patient handoff procedures in virtual health, discharge planning, transfer protocols, and strategies to ensure continuity of care during transitions
	C50. Knowledge of strategies to ensure continuity of care when transitioning to virtual health services.
	C51. The ability to detect and effectively coordinate care with various providers involved in the patient care process
	C52. The ability to identify and coordinate care between providers on the patient's care team in virtual healthcare
	C53. Determine the need and timing of referral to another specialist in or out of the virtual care settings.
Domain. 9	**Cultural competency in virtual healthcare**
	C54. Understanding and recognizing diverse populations' cultural beliefs, values, practices, and norms. This includes awareness of cultural backgrounds, languages, religions, and customs.
	C55. Knowledge of various languages spoken by different cultural groups bei, ng aware of potential language barriers, and using interpreters or translation services in virtual healthcare when necessary
	C56. The ability to work effectively with patients from diverse cultural backgrounds and adapt to their needs in virtual healthcare
	C57. Developing an attitude of respect, empathy, and sensitivity toward patients' cultural differences in virtual Healthcare
Domain10	**Data Analytics and Interpretation in Virtual Healthcare**
	C58. Familiarity with the various sources of virtual health data, such as electronic health records (EHRs), wearables, mobile apps, and virtual health platforms
	C59. Proficiency in statistical methods and techniques used for analyzing healthcare data in virtual healthcare, including descriptive statistics, inferential statistics, hypothesis testing, regression analysis, and survival analysis
	C60. Knowledge of data visualization tools and techniques to present healthcare data in virtual healthcare clearly and understandably. This includes creating charts, graphs, and dashboards for effective communication and decision-making.
	C61. The ability to visualize and interpret diagnostic tests and imaging results remotely
	C62. The ability to analyze and interpret the information provided through virtual platforms quickly to make informed decisions during procedures, for example, remote patient monitoring data
Domain 11	**Disease-Specific Management in Virtual Healthcare**
	C63. Knowledge of various diseases managed virtually, including their causes, symptoms, diagnosis, treatment protocols, potential complications, and follow-up care requirements. This understanding enables accurate diagnosis and effective treatment planning in virtual healthcare.
	C64. Proficiency in diagnostic techniques and tools used to interpret clinical findings, analyze test results, and make accurate diagnoses in virtual healthcare.
	C65. Knowledge of disease management strategies, including medication management, lifestyle modifications, and self-care techniques that can be implemented through virtual platforms

	C66. The ability to conduct rapid assessment of stroke symptoms via video, prompt diagnosis and treatment decisions, collaboration with neurologists and stroke teams, remote monitoring of vitals and neurological status
	C67. The ability to conduct remote EKG interpretation, cardiac auscultation using specialized virtual health equipment, remote monitoring of heart rhythm and vital signs, managing chronic heart conditions like arrhythmias and heart failure, patient education on heart health and medication adherence
	C68. The ability to conduct virtual consultations for cancer patients, provide emotional support and guidance, manage treatment side effects remotely, monitor progress and adjust treatment plans, collaborate with oncologists and other specialists
	C69. The ability to manage chronic conditions common in older adults, remote assessment of cognitive function and mental health, fall prevention education and monitoring, medication management, and adherence, addressing social isolation and loneliness
Domain 12	**Recordkeeping and Documentation in Virtual Healthcare**
	C70. Knowledge of medical terminology in virtual healthcare to accurately document patient information and communicate effectively with other professionals.
	C71. Understanding and adhering to documentation standards and electronic health records and documentation in virtual care settings
	C72. The ability to maintain accurate, clear, and complete electronic health records and securely manage patient data in virtual healthcare
	C73. Accurately documenting virtual consultations and maintaining proper records.
	C74. Ensuring patient information is kept secure and maintaining confidentiality
Domain13	**Effective Communication in Virtual Healthcare**
	C75. Knowledge of the essential role of communication in virtual care and the different types of communication
	C76. The ability to promote effective communication within the team in virtual healthcare, providing feedback and support to staff who need assistance to develop communication skills
	C77. The ability to demonstrate practical verbal and non-verbal communication skills to promote patient safety in virtual healthcare
	C78. Ensure the information being conveyed is easily understood by patients or colleagues via virtual healthcare.
	C79. Recognize and understand the emotions of patients or colleagues in virtual healthcare and respond appropriately, offering support and reassurance when necessary.
	C80. Introduce yourself, your role, and your organization, and obtain consent for any interventions.
Domain14	**Emergency response and crisis management in virtual healthcare**
	C81. Knowledge of virtual health disaster response and emergency preparedness
	C82. Understanding crisis management strategies applicable to virtual healthcare settings.
	C83. Knowledge of backup platforms and communication channels that will be used in case of technical failures or emergencies. Healthcare professionals should be prepared to switch to alternative methods for maintaining patient contact and critical communication.
	C84. The ability to assess the severity of the crisis, categorize patients based on urgency, and determine appropriate and instant emergency care that may arise and address the critical technical problems and risk management
	C85. Recognizing the importance of proactive crisis response planning to ensure the continuity and safety of virtual health services.

	C86. Calm and composed attitude during crises, recognizing the need for effective crisis management to ensure patient safety and well-being.
	C87. Commitment to attending regular training in emergency response protocols, crisis management techniques, and virtual tools utilization
Domain15	**Research development and Evidence-Based Practice Utilization in Virtual Healthcare**
	C88. Understand the principles of evidence-based practice and how to integrate the best available evidence into the virtual health decision-making process.
	C89. The ability to identify and acquire relevant research articles and studies in virtual healthcare. This includes proficiently searching medical databases, critically appraising research literature, and understanding statistical analysis.
	C90. The ability to critically appraise research and evaluate the quality and validity of research studies. This involves assessing the study design, methodology, sample size, statistical analyses, and the relevance of the findings to the clinical question.
	C91. Commitment to staying current with new research developments and advancements and continually updating clinical practice based on the best available evidence
Domain 16	**Governance, risk, and quality management in virtual healthcare**
	C92. Knowledge of local, national, and international virtual health standards to ensure compliance with laws and regulations in healthcare.
	C93. Understanding the principles of risk management in virtual healthcare and outcome measures
	C94. The ability to lead or participate in quality improvement initiatives to enhance patient outcomes and satisfaction in virtual healthcare settings
	C95. The ability to identify potential risks, evaluate their impact on patient safety and quality of care, and implement strategies to mitigate those risks in virtual healthcare settings
	C96. A commitment to continuous improvement fosters accountability and dedication to providing high-quality virtual health services.
Domain.17	**Health Information Management in Virtual Healthcare**
	C97. Proficiency in various health information systems and technologies used in virtual healthcare for storing, organizing, and retrieving health data
	C98. Understand key information technology concepts and components in virtual healthcare, such as networks, storage devices, operating systems, and applications.
	C99. Familiarity with health information exchange standards and protocols to enable effective sharing and exchange of health information between different healthcare organizations and systems
	C100. Understanding the principles and practices of information governance, including data quality, data integrity, and data standards, to maintain accurate and reliable health information in virtual healthcare
	C101. The ability to evaluate and recommend reliable digital health resources, applications, and information for patient education and self-management
	C102. Express the ability to utilize informatics in virtual healthcare settings.
Domain 18	**Data security and privacy in virtual healthcare**
	C103. Comprehensive understanding of the privacy, confidentiality, security, and safety features of virtual health tools
	C104. The ability to maintain privacy and confidentiality and comply with security regulations during virtual consultations
	C105. The ability to ensure that patient data is protected from unauthorized access, modification, or disclosure in virtual healthcare. This involves implementing encryption, secure data storage, and access control mechanisms.

	C106. The ability to obtain patient consent in virtual healthcare and authorization before collecting, using, or disclosing their health information.
	C107. Respect and acknowledgment of patients' confidentiality and autonomy throughout the virtual health delivery
Domain.19	**Professional Development in Virtual Healthcare**
	C108. Updated Clinical Knowledge by continuously updating knowledge and skills to keep up with advancements in clinical and technology
	C109. Engaging in professional development activities and seeking feedback to improve clinical and virtual healthcare skills continuously
	C110. The ability to identify personal learning needs and focus on attending the necessary training to equip them with current knowledge and skills relevant to virtual health technologies.
	C111. The ability to educate and train other healthcare professionals in virtual health
	C112. Willing to learn and undertake more studies throughout their professional lives in virtual healthcare
	C113. Participate actively in activities like workshops and conferences relevant to virtual health.
	C114. Show a commitment to personal growth and self-development by engaging in activities that increase their competency, such as networking with other professionals and continuing with education.
Domain.20	**Patient Assessment and Diagnosis in Virtual Healthcare**
	C115. Knowledgeable in gathering a patient's medical history remotely, including previous illnesses, family history, lifestyle factors, and medication history.
	C116. The ability to assess, plan care, support, and manage treatment using recognized tools in clinical and virtual settings
	C117. The ability to guide patients to self-perform certain examinations, such as observing skin conditions, checking vital signs with home devices, or assessing range of motion through guided movements
	C118. The ability to engage patients in their care through virtual channels and motivate them to participate in their health management.
Domain 21	**Patient education and engagement in virtual healthcare**
	C119. The ability to effectively educate and guide patients remotely on self-management techniques, exercises, and preventative measures to promote long-term rehabilitation and well-being
	C120. The ability to provide clear and concise instructions and educate patients about their conditions, treatments, and self-care
Domain 22	**Patient Safety in Virtual Healthcare**
	C121. Comprehensive understanding of safety protocols, guidelines, and patient safety culture, including reporting and learning from errors, engaging in continuous quality improvement, and advocating for patient safety
	C122. The ability to lead and manage patient safety initiatives, promoting a culture of safety in virtual care within their organizations
	C123. Participate actively in quality assurance programs, incident reporting systems, and ongoing monitoring of patient outcomes, contributing to a culture of continuous improvement.
Domain 23	**Patient-Centered care in virtual healthcare**
	C124. The ability to engage and involve patients and their families in virtual health decision-making processes
	C125. The ability to identify and provide patient-centered, family-focused care that understands and respects diversity and inclusion in virtual healthcare settings
	C126. The ability to empathize with patients, understanding their emotions, fears, and concerns in virtual healthcare settings
	C127. Commitment to patient-centered care
	C128. Respect and support clients' right to make informed decisions about their care

Domain 24	**Remote Medication Management**
	C129. Understanding of medication safety protocols and best practices to minimize errors during remote prescribing and medication administration
	C130. In-depth knowledge of different medications that can be prescribed remotely, including their indications, interactions, contraindications, dosing, side effects, and potential adverse reactions
	C131. The ability to review the patient's medical history, medication lists from different providers, and laboratory results to confirm medication accuracy and identify potential interactions or contraindications
	C132. It is crucial to remote medication management to educate patients about their medications remotely, including proper usage, potential side effects, and adherence.
	C133. The ability to prescribe medications remotely, assessing medication adherence, and providing virtual medication management
Domain 25	**Remote Patient Monitoring**
	C134. Understanding of the conditions being monitored and the relevant physiological parameters.
	C135. Familiarity with the remote monitoring technology and devices being used, such as wearable devices, mobile apps, and virtual health platforms and their applications in healthcare
	C136. The ability to monitor patients remotely and respond to changes in their condition promptly
	C137. Emphasizing the importance of leveraging technology for continuous patient health assessment and proactive monitoring
	C138. A commitment to keep updated with developments in RPM technology, data analysis tools, and best practices to continue providing optimal care for patients in virtual healthcare settings.
Domain 26	**Public and Community Health in Virtual Healthcare**
	C139. Understanding community dynamics, social determinants of health, and community to identify health needs and develop interventions that empower individuals and communities in virtual healthcare.
	C140. Knowledgeable about various national prevention strategies and programs, such as immunizations, screening programs, and infection control practices
	C141. The ability to interpret health indicators, track trends, and contribute to research studies to improve population health outcomes in virtual healthcare.
	C142. The ability to assess and manage public health concerns through virtual health interventions
	C143. Recognizing the role of virtual health interventions in contributing to broader population health outcomes and public health advocacy.
	C144. A commitment to promoting public health in virtual settings, recognizing the broader impact of virtual healthcare on population well-being.
Domain 27	**The Use of Artificial Intelligence**
	C145. Understanding the range of health-related AI applications, including their capabilities, limitations, and potential applications in their specialty in virtual healthcare settings.
	C146. The ability to interpret AI-generated insights and incorporate them into clinical and virtual decision-making processes.
Domain.28	**Innovation and Creativity in Virtual Healthcare**
	C147. Understanding the adoption and integration of innovative technologies in virtual healthcare
	C148. Have the skills to share innovative ideas and develop creative solutions in a virtual health setting.
	C149. Be able to commit to Identifying innovative methods that are cost-effective and sustainable.
Domainn29	**Project Management in Virtual Healthcare**
	C150. Ability to plan and manage projects by defining project scope, having clear objectives, setting timelines, managing resources, and ensuring deliverables are met on time and within budget.

	C151. The ability to set a well-defined project by identifying the project's objectives, scope, deliverables, and schedule.
Domain 30	**Marketing and Outreach of Virtual Health**
	C152. Ability to market and reach the strategies needed to promote virtual health services.
	C153. Advocating the benefits of virtual care
	C154. Ability to use patient testimonials, success stories, and case studies to showcase the advantages of virtual healthcare
	C155. Ability to acknowledge the role of marketing and outreach to increase awareness and utilize the services of virtual health
Domain 31	**Virtual Health Policy Development and Advocacy**
	C156. Recognize the existing policies and guidelines related to virtual health to help identify gaps.
	C157. Ability to identify healthcare policy issues and come up with strategies that are relevant to virtual health.
	C158. Have skills to influence the Development of policies and foster favorable regulatory environments for virtual health
	C159. Understand the need to actively engage with policy discussions to shape the regulatory setting of virtual healthcare.
Domain 32	**Billing and coding in virtual healthcare**
	C160. Knowledge of virtual health financial models, reimbursement mechanisms, and budgeting.
	C161. Understand billing codes, health insurance, and coverage in virtual healthcare settings.
	C162. The ability to understand virtual healthcare's billing and coverage aspects and manage resources effectively
Domain 33	**Cybersecurity in Virtual Healthcare**
	C163. Understand network security fundamentals, including firewalls, intrusion detection and prevention systems, and secure configuration management.
	C164. Knowledge of the basics of cybersecurity, including safe browsing, email security, and recognizing social engineering attempts
	C165. The ability to protect patient information and maintain cybersecurity in a virtual environment
